# Longitudinal prevalence of potentially inappropriate medicines and potential prescribing omissions in a cohort of community-dwelling older people

**DOI:** 10.1007/s00228-015-1815-1

**Published:** 2015-02-11

**Authors:** Frank Moriarty, Kathleen Bennett, Tom Fahey, Rose Anne Kenny, Caitriona Cahir

**Affiliations:** 1 HRB Centre for Primary Care Research, Department of General Practice, Royal College of Surgeons in Ireland, 123 St Stephens Green, Dublin 2, Ireland; 2 Department of Pharmacology & Therapeutics, Trinity Centre for Health Sciences, St James’s Hospital, Dublin 8, Ireland; 3 The Irish Longitudinal Study on Ageing, Trinity College Dublin, Dublin 2, Ireland

**Keywords:** STOPP, START, Beers criteria, ACOVE indicators, Cohort, Potentially inappropriate prescribing

## Abstract

**Purpose:**

This study aims to compare the prevalence of potentially inappropriate medicines (PIMs) and potential prescribing omissions (PPOs) using several screening tools in an Irish community-dwelling older cohort, to assess if the prevalence changes over time and to determine factors associated with any change.

**Methods:**

This is a prospective cohort study of participants aged ≥65 years in The Irish Longitudinal Study on Ageing (TILDA) with linked pharmacy claims data (*n* = 2051). PIM and PPO prevalence was measured in the year preceding participants’ TILDA baseline interviews and in the year preceding their follow-up interviews using the Screening Tool for Older Persons’ Prescriptions (STOPP), Beers criteria (2012), Assessing Care of Vulnerable Elders (ACOVE) indicators and the Screening Tool to Alert doctors to Right Treatment (START). Generalised estimating equations were used to determine factors associated with change in prevalence over time.

**Results:**

Depending on the screening tool used, between 19.8 % (ACOVE indicators) and 52.7 % (STOPP) of participants received a PIM at baseline, and PPO prevalence ranged from 38.2 % (START) to 44.8 % (ACOVE indicators), while 36.7 % of participants had both a PIM and PPO. Common criteria were aspirin for primary prevention (19.6 %) and omission of calcium/vitamin D in osteoporosis (14.7 %). Prevalence of PIMs and PPOs increased at follow-up (PIMs range 22–56.1 %, PPOs range 40.5–49.3 %), and this was associated with patient age, female sex, and numbers of medicines and chronic conditions.

**Conclusions:**

Sub-optimal prescribing is common in older patients. Ongoing prescribing review to optimise care is important, particularly as patients get older, receive more medicines or develop more illnesses.

**Electronic supplementary material:**

The online version of this article (doi:10.1007/s00228-015-1815-1) contains supplementary material, which is available to authorized users.

## Introduction

Medicines are the most common healthcare intervention worldwide, and despite providing many benefits, they also carry potential risks which can lead to patient harm [1]. Physiological changes in ageing which alter drug pharmacodynamics and pharmacokinetics can predispose older people to adverse drug events [2]. Additionally, older people are more likely to be taking multiple medicines and have multiple medical conditions, increasing the likelihood of drug-drug or drug-disease interactions [2–4].

Due to concerns regarding appropriate medication use in this age group, a number of screening tools/criteria have been devised to define what constitutes potentially inappropriate prescribing (PIP) in the elderly. PIP can be classified as either (i) potentially inappropriate medicines (PIMs), the use of a medicine where no clear clinical indication exists or the use of an indicated medicine in circumstances where the risks outweigh the benefits, or (ii) potential prescribing omissions (PPOs), not prescribing a beneficial medicine for which there is a clear clinical indication [5].

Although PIP can be determined implicitly on the basis of clinician’s judgement, the majority of research has determined it explicitly using published criteria/screening tools, a large number of which have been developed [5]. The earliest such tool was Beers criteria, first published in 1991 as a list of drugs to be avoided in older nursing home residents [6]. It contained many medicines not commonly prescribed outside of the USA, and updates to the Beers criteria include drugs which are more widely used internationally and also drugs to avoid with certain conditions [7]. Although earlier iterations of the Beers criteria have been used extensively in the literature, the 2012 criteria have yet to be widely applied [8]. The Screening Tool for Older Person’s Prescriptions (STOPP) and Screening Tool to Alert doctors to Right Treatment (START) were developed as screening tools for PIMs and PPOs, respectively, suitable for use in European countries and have been applied and validated in the literature [9–12]. Outside of specific PIP screening tools, the Assessing Care of Vulnerable Elders (ACOVE) indicators were developed by the Research and Development (RAND) Corporation to assess the overall quality of care of older people [13]. Several ACOVE indicators relate to PIMs and PPOs, and these have been assessed for use as a PIP screening tool and have good inter-rater reliability [14, 15].

Much published literature on this topic has only focussed on PIMs with few studies utilising PPO screening tools. Of those studies that considered both PIMs and PPOs, none have reported how these two forms of PIP overlap [16–21]. Additionally, little is known about how the prevalence of PIMs and PPOs changes over time in older populations and determinants of change. This study aims to (i) compare the prevalence of potentially inappropriate prescribing (both PIMs and PPOs) in a cohort of community-dwelling people aged 65 years and older in Ireland according to a number of screening tools and (ii) assess if the prevalence of potentially inappropriate prescribing in this cohort changes over time and to determine the factors associated with any change.

## Methods

### Study population

The population for this prospective cohort study comprised a subset of participants in The Irish Longitudinal Study on Ageing (TILDA) for whom medication dispensing history was available. TILDA is a nationally representative cohort study of more than 8000 community-dwelling people aged 50 years or over charting their health, social, and economic circumstances (see Supplementary file 1 for details of collected information). TILDA participants were deemed eligible for this study if they were aged 65 years or older at baseline and supplied a valid General Medical Services (GMS) identifier to allow linkage of their medication dispensing history from the Health Service Executive Primary Care Reimbursement Service (HSE-PCRS) pharmacy claims database. The HSE-PCRS GMS scheme provides free health services to eligible persons in Ireland, including a wide range of prescribed medicines, although a monthly co-payment per prescription item has applied since October 2010. Eligibility for the GMS scheme is based on means testing, although all people aged over 70 were eligible until December 2008 when a higher income threshold was introduced for this age group compared to the general population.

### Data collection

TILDA participant recruitment and wave 1 baseline data collection were carried out between 2009 and 2011 when participants were interviewed face to face, completed a questionnaire and underwent a health assessment. TILDA follow-up waves are scheduled every 2 years, the first of which was carried out from February 2011 to March 2012. Medication data were extracted from the HSE-PCRS pharmacy claims database on the basis of GMS identifier for each participant in the present study for the 15 months preceding the date of their TILDA baseline and follow-up interviews. The HSE-PCRS database contains a GMS identifier, sex and date of birth, drug information (World Health Organisation Anatomical Therapeutic Chemical (ATC) code, strength, defined daily dosage (DDD)), quantity dispensed and date of dispensing. Linkage of participants’ TILDA and HSE-PCRS dispensing data was carried out using methods published previously [22]. All data were anonymised after linkage was performed. Ethical approval for TILDA was provided by the Faculty of Health Sciences Ethics Committee at Trinity College Dublin.

### Potentially inappropriate prescribing criteria

The prevalence of PIP was measured during two time intervals: in the 12 months preceding each participant’s baseline TILDA interview and in the 12 months preceding their follow-up interview. A longer period of time was analysed for criteria dependent on duration of medication use of greater than 1 month to allow for 12 months of potential exposure. Dispensing data from the HSE-PCRS database and information from TILDA on diagnoses, medications not included in the HSE-PCRS database (self-reported) and other characteristics were used to assess if participants had received a PIM or had a PPO. Participants were classified as having a PIM if they were prescribed the potentially inappropriate medicine at any time during the study periods, while having a PPO was classified as not receiving the indicated medicine at any time during the study periods.

A subset of criteria from STOPP, Beers criteria (2012) and the third iteration of the ACOVE indicators were applied to dispensing data and information from TILDA to measure PIMs. Forty-five of 65 (69 %) STOPP criteria, 42 of 52 (81 %) Beers criteria and 17 of 22 (77 %) ACOVE indicators relating to inappropriate medicines were used. To measure PPOs, a subset of criteria from START and the ACOVE indicators were used. Fifteen of 22 (68 %) START criteria were applicable, while 21 of 65 (34 %) ACOVE indicators relating to prescribing indicated that medicines could be applied. All criteria for which the necessary participant information was available in the HSE-PCRS pharmacy claim that database and TILDA were applied.

### Statistical analysis

Prevalence estimates were calculated for each set of PIM and PPO criteria as well as for each individual criterion. Additionally, the prevalence of each criterion (where applicable) as a proportion of the number of participants with the condition/disease or prescribed the drug of interest was determined, for example, of those prescribed a benzodiazepine during the study period, the proportion who were prescribed it for >4 weeks. McNemar’s test for paired groups comparison was used to test whether the prevalence of criteria changed significantly between the two time periods. Generalised estimating equations (GEE) with exchangeable correlations were used to investigate determinants of the change in prevalence of PIMs and PPOs [23]. Unadjusted analysis estimating change in overall PIM and PPO prevalence from baseline to follow-up was followed by multivariable GEE analysis which adjusted for sex, age, numbers of regular medicines and diagnosed chronic conditions (reported at TILDA interview) at baseline and follow-up in the models. Level of educational attainment as an indicator of socio-economic status was assessed for inclusion in the models. Analyses were performed using SAS 9.2 (SAS Institute Inc., Cary, NC, USA) and Stata version 12 (Stata Corporation, College Station, TX, USA).

## Results

### Sample characteristics

**Fig. 1:**
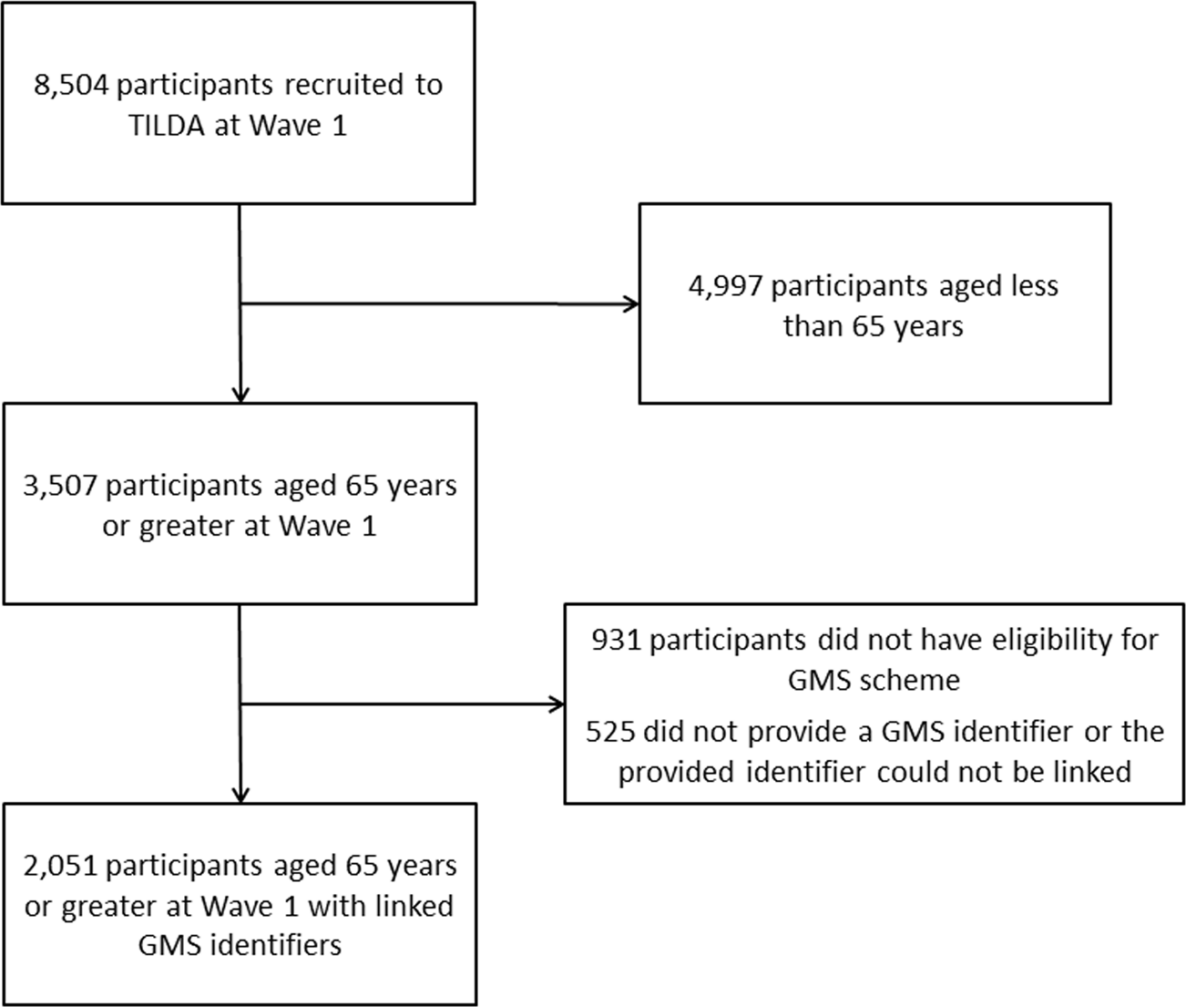
Flow diagram of study participants from TILDA cohort

This study included 2051 TILDA participants (Fig. 1), of which 1107 (53.97 %) were female, and mean participant age (SD) in this sample was 74.8 (6.2) years. Mean number of reported regular medicines (SD) increased from 4.1 (2.9) at baseline to 4.9 (3.2) at follow-up, while mean number of chronic conditions reported (SD) was 2.4 (1.6) and 2.9 (1.7) at TILDA baseline and follow-up interviews, respectively.

### Prevalence of potentially inappropriate medicine use and potential prescribing omissions

When assessed using STOPP, 1081 participants (52.7 %) were prescribed a PIM during the baseline study period, while prevalence of Beers PIMs and ACOVE PIMs was significantly lower (30.5 and 19.8 % respectively, *p* < 0.05). The prevalence of PPOs at baseline was 44.8 % when assessed using ACOVE indicators and 38.2 % using START (see Table 1). Overall, 61.4 % of the sample had a PIM defined by any of the screening tools, 53.3 % had any PPO and 753 (36.7 %) participants had both a PIM and PPO. A total of 2963 PIMs and 2315 PPOs were identified during this study period.

**Table 1:** Number of participants with PIMs and PPOs at baseline and 2-year follow-up

Screening tool	Baseline		Follow-up	
	*N*	% (95 % CI)	*N*	% (95 % CI)
STOPP	1080	52.7 (50.5, 54.8)	1151	56.1 (54.0, 58.3)
1	611	29.8 (27.8, 31.8)	602	29.4 (27.4, 31.3)
2	270	13.2 (11.7, 14.6)	307	15.0 (13.4, 16.5)
≥ 3	200	9.8 (8.5, 11.0)	242	11.8 (10.4, 13.2)
Beers criteria	625	30.5 (28.5, 32.5)	678	33.1 (31.0, 35.1)
1	325	15.8 (14.3, 17.4)	349	17.0 (15.4, 18.6)
2	192	9.4 (8.1, 10.6)	203	9.9 (8.6, 11.2)
≥ 3	108	5.3 (4.3, 6.2)	126	6.1 (5.1, 7.2)
ACOVE indicators	407	19.8 (18.1, 21.6)	451	22.0 (20.2, 23.8)
1	336	16.4 (14.8, 18.0)	372	18.1 (16.5, 19.8)
2	62	3.0 (2.3, 3.8)	63	3.1 (2.3, 3.8)
≥ 3	9	0.4 (0.2, 0.7)	16	0.8 (0.4, 1.2)
Any above PIM^a^	1259	61.4 (59.3, 63.5)	1330	64.8 (62.8, 66.9)
START	783	38.2 (36.1, 40.3)	831	40.5 (38.4, 42.6)
1	551	26.9 (24.9, 28.8)	586	28.6 (26.6, 30.5)
2	171	8.3 (7.1, 9.5)	171	8.3 (7.1, 9.5)
≥ 3	61	3.0 (2.2, 3.7)	74	3.6 (2.8, 4.4)
ACOVE indicators	918	44.8 (42.6, 46.9)	1011	49.3 (47.1, 51.5)
1	465	22.7 (20.9, 24.5)	494	24.1 (22.2, 25.9)
2	279	13.6 (12.1, 15.1)	331	16.1 (14.5, 17.7)
≥ 3	174	8.5 (7.3, 9.7)	186	9.1 (7.8, 10.3)
Any above PPO^a^	1094	53.3 (51.2, 55.5)	1161	56.6 (54.5, 58.8)

^a^PIM and PPO screening tools are not mutually exclusive, overall prevalence of PIMs and PPOs accounts for any overlap

The most common (prevalence >2 %) individual PIM criteria and PPO criteria are reported in Tables 2 and 3, respectively. All applied criteria are presented in Supplementary file 2. The most prevalent baseline PIM criteria were aspirin with no history of coronary, cerebral or peripheral arterial symptoms or occlusive arterial event (STOPP, 19.6 %) and proton pump inhibitor (PPI) at full therapeutic dosage for >8 weeks (STOPP, 17.2 %). The most prevalent baseline PPO criteria were calcium and vitamin D supplement omission in patients with self-reported osteoporosis (ACOVE indicators and START, 14.7 %) and omission of a laxative in an older person with persistent pain treated with opioids (ACOVE indicators, 11.0 %).

**Table 2:** Prevalence of individual PIM criteria (prevalence ≥2 %) at baseline and 2-year follow-up

Criteria description	Baseline			Follow-up			
	*n*	% of sample	% of indication^a^	*n*	% of sample	% of indication^a^	Change in prevalence (95 % CI)
STOPP							
Cardiovascular system							
Loop diuretic for dependent ankle oedema only	97	4.7	–	114	5.6	–	0.8 (−0.6, 2.2)
Aspirin with history of PUD without H_2_ receptor antagonist or PPI	40	2.0	71.4	38	1.9	59.4	−0.1 (−0.9, 0.7)
Aspirin with no history of coronary, cerebral or peripheral arterial symptoms or occlusive arterial event	402	19.6	–	377	18.4	–	−1.2 (−3.9, 1.4)
Central nervous system							
TCAs with an opiate or calcium channel blocker	46	2.2	–	69	3.4	–	1.1 (0.1, 2.1)**
Long-term (>1 month), long-acting benzodiazepines	80	3.9	34.8	64	3.1	33.0	−0.8 (−1.9, 0.4)*
Gastrointestinal System							
PPI at full therapeutic dosage for >8 weeks	353	17.2	42.6	450	21.9	47.4	4.7 (2.0, 7.4)***
Musculoskeletal system							
NSAID with history of PUD, unless with concurrent H_2_ receptor antagonist, PPI or misoprostol	37	1.8	77.1	41	2.0	83.7	0.2 (−0.6, 1.0)
NSAID with moderate-severe hypertension >160/100 mmHg^b^	187	9.1	33.0	207	10.1	34.0	1.0 (−0.9, 2.9)
Long-term use of NSAID (>3 months)	99	4.8	14.2	107	5.2	16.2	0.4 (−1.0, 1.8)
Long-term corticosteroids (>3 months) as monotherapy for rheumatoid arthrtitis/osteorarthritis	37	1.8	20.6	47	2.3	20.8	0.5 (−0.4, 1.4)
Drugs that adversely affect fallers							
Benzodiazepines in those prone to falls	63	3.1	14.4	55	2.7	11.4	−0.4 (−1.4, 0.6)
Neuroleptic drugs in those prone to falls	34	1.7	7.8	49	2.4	10.2	0.7 (−0.1, 1.6)
Analgesic drugs							
Regular opiates for >2 weeks without concurrent use of laxatives	109	5.3	90.8	103	5.0	84.4	−0.3 (−1.7, 1.1)
Duplicate drug classes							
Any regular duplicate drug class prescription	51	2.5	–	66	3.2	–	0.7 (−0.3, 1.8)
Beers criteria (2012)							
Anticholinergics							
Antispasmodics	35	1.7	–	46	2.2	–	0.5 (−0.3, 1.4)
Central nervous system							
Tertiary TCAs	97	4.7	–	119	5.8	–	1.1 (−0.3, 2.5)*
Benzodiazepines, short, intermediate and long acting	201	9.8	–	180	8.8	–	−1.0 (−2.9,0.8)
Non-benzodiazepine (Z-drug) hypnotics, avoid chronic use >90 days	48	2.3	17.3	56	2.7	17.4	0.4 (−0.6, 1.4)
Gastrointestinal							
Metoclopramide	22	1.1	–	42	2.0	–	1.0 (0.2, 1.7)*
Pain							
Non-COX-selective NSAIDs, avoid chronic use	93	4.5	12.1	81	3.9	10.6	−0.6 (−1.8, 0.7)
Drug-disease interactions							
Avoid with history of falls/fractures (fracture and fall or > 1 fall or >1 fracture) (total)	124	6.0	54.4	168	8.2	59.2	2.1 (0.5, 3.8)***
Anticonvulsants	20	1.0	8.8	46	2.2	16.2	1.3 (0.5, 2.0)***
Benzodiazepines	64	3.1	28.1	76	3.7	26.8	0.6 (−0.5, 1.7)
Z-drugs	42	2.0	18.4	60	2.9	21.1	0.9 (−0.1, 1.8)
SSRIs	40	2.0	17.5	45	2.2	15.8	0.2 (−0.6, 1.1)
ACOVE indicators							
Falls and mobility problems							
If ≥2 falls (or 1 fall with injury) in previous year discontinue benzodiazepine	43	2.1	14.9	44	2.1	12.0	0.0 (−0.8, 0.9)
Hypertension							
If a vulnerable elder (VE) has HTN discontinue NSAID or COX-2 inhibitor^b^	79	3.9	7.8	80	3.9	7.1	0.0 (−1.2, 1.3)
Medication Use							
Discontinue benzodiazepine if taking for >1 month	80	3.9	34.8	64	3.1	33.0	−0.8 (−1.9, 0.4)*
Avoid medication with strong anticholinergic effects	245	11.9	–	288	14.0	–	2.1 (−0.1, 4.3)**
In iron-deficiency anaemia, prescribe no more than one low-dose oral iron tablet daily	27	1.3	20.1	43	2.1	30.3	0.8 (0.0, 1.6)

*ACE* angiotensin converting enzyme, *COPD* chronic obstructive pulmonary disease, *COX* cyclo-oxygenase, *GI* gastrointestinal, *HTN* hypertension, *MI* myocardial infarction, *NSAID* non-steroidal anti-inflammatory drug, *PPI* proton pump inhibitor, *PUD* peptic ulcer disease, *SSRI* selective serotonin reuptake inhibitor, *TCA* tricyclic antidepressant, *TIA* transient ischaemic attack, *VE* vulnerable elder

*McNemar’s test *p* < 0.05

**McNemar’s test *p* < 0.01

***McNemar’s test *p* < 0.001

^a^Prevalence of PIM criteria as a proportion of all participants with the disease or prescribed the drug of interest e.g. prevalence of benzodiazepines for >4 weeks as a proportion of all participants prescribed a benzodiazepine

^b^Hypertension defined using objectively measured blood pressure or self-reported hypertension diagnosis with antihypertensive medication

**Table 3:** Prevalence of individual PPO criteria (prevalence ≥2 %) at baseline and 2-year follow-up

	Baseline			Follow-up			
Criteria description	*n*	% of sample	% of indication^a^	*n*	% of sample	% of indication^a^	Change in prevalence (95 % CI)
START							
Cardiovascular system							
Warfarin (or another oral anticoagulant) in the presence of chronic atrial fibrillation	154	7.5	67.5	190	9.3	62.9	1.8 (0.1, 3.4)***
Aspirin/clopidogrel with a history of atherosclerotic coronary, cerebral or peripheral vascular disease	48	2.3	14.2	51	2.5	14.6	0.1 (−0.7, 1.0)
Antihypertensive therapy where systolic blood pressure >160 mmHg^b^	77	5.5	31.3	49	3.5	19.6	−2.0 (−3.4, −0.6)***
Statin therapy with a history of coronary, cerebral or peripheral vascular disease	67	3.3	20.7	82	4.0	21.8	0.7 (−0.3, 1.8)*
ACE inhibitor following acute MI	61	3.0	14.6	58	2.8	11.9	−0.1 (−1.0, 0.8)
β blocker with chronic stable angina	84	4.1	37.3	81	3.9	33.8	−0.1 (−1.2, 0.9)
Respiratory system							
Regular inhaled β2 agonist or anticholinergic agent for mild to moderate asthma or COPD	102	5.0	37.0	116	5.7	37.5	0.7 (−0.6, 2.0)
Musculoskeletal system							
Bisphosphonates if taking oral corticosteroids for >3 months	62	3.0	60.2	71	3.5	64.5	0.4 (−0.5, 1.4)
Calcium and vitamin D supplement with osteoporosis	301	14.7	57.9	329	16.0	51.6	1.4 (−0.9, 3.6)*
Endocrine system							
Antiplatelet therapy in diabetes mellitus if ≥1 major CV risk factor (hypertension, hypercholesterolaemia, smoking history)	56	2.7	27.6	64	3.1	28.6	0.4 (−0.5, 1.4)
Statin therapy in diabetes mellitus if ≥1 major CV risk factor	50	2.4	24.6	50	2.4	22.3	1.4 (−0.9, 3.6)
ACOVE indicators							
COPD							
If a VE has COPD, prescribe a rapid-acting bronchodilator	43	2.1	40.2	66	3.2	46.2	1.1 (0.3, 2.0)***
If a VE with COPD has 2+ exacerbations requiring antibiotics/oral corticosteroids in the previous year, then (in addition to a long-acting bronchodilator) prescribe inhaled steroids (if not taking oral steroids)	20	1.0	58.8	41	2.0	63.1	1.0 (0.4, 1.6)
Diabetes							
If a VE with diabetes mellitus not on anticoagulant or antiplatelet, then daily aspirin should be prescribed	54	2.6	26.9	58	2.8	27.2	0.2 (−0.7, 1.1)
Hypertension							
If a VE with HTN has IHD, prescribe a β blocker^c^	62	3.0	37.1	63	3.1	34.6	0.0 (−0.9, 1.0)
If a VE with HTN has a history of HF, IHD, chronic kidney disease or CV accident, prescribe an ACE inhibitor/ARB^c^	66	3.2	29.2	68	3.3	26.8	0.1 (−0.9, 1.1)
Ischaemic heart disease							
If a VE has had an MI, prescribe a β blocker	61	3.0	34.5	59	2.9	30.3	−0.1 (−1.0, 0.8)
If a VE has IHD, prescribe an ACE inhibitor/ARB	81	3.9	36.0	82	4.0	34.2	0.0 (−1.0, 1.1)
Medication use							
If a VE with a risk factor for GI bleeding (aged ≥75, PUD, warfarin use, chronic glucocorticoid use) is prescribed a non-selective NSAID, treat concomitantly with misoprostol/a PPI	207	10.1	68.5	182	8.9	65.0	−1.2 (−3.0, 0.5)
Osteoporosis							
If a VE without osteoporosis is taking ≥7.5 mg/day of prednisone (or equivalent) for ≥1 month, prescribe calcium and vitamin D	53	2.6	59.6	53	2.6	57.6	0.0 (−0.8, 0.8)
If a VE has osteoporosis, prescribe calcium and vitamin D supplements	301	14.7	57.9	329	16.0	51.6	1.4 (−0.9, 3.6)
If a female VE has osteoporosis, treat with bisphosphonate, raloxifene, calcitonin, HRT or teriparatide	186	9.1	48.9	249	12.1	53.2	3.1 (1.2, 4.9)***
If a male VE has osteoporosis, treat with bisphosphonate, calcitonin, parathyroid hormone or testosterone	123	6.0	87.9	144	7.0	85.2	1.0 (−0.4, 2.5)***
Pain							
If a VE with persistent pain is treated with opioids, prescribe a stool softener/laxative	225	11.0	82.7	265	12.9	82.0	2.0 (0.0, 3.9)*
Stroke							
If a VE has had a TIA or stroke, prescribe antiplatelet/anticoagulant therapy	83	4.0	58.9	99	4.8	54.4	0.8 (−0.4, 1.9)**

*ACE* angiotensin converting enzyme, *ARB* angiotensin II receptor blocker, *COPD* chronic obstructive pulmonary disease, *COX* cyclo-oxygenase, *CV* cardiovascular, *GI* gastrointestinal, *HF* heart failure, *HRT* hormone replacement therapy, *HTN* hypertension, *IHD* ischaemic heart disease, *MI* myocardial infarction, *NSAID* non-steroidal anti-inflammatory drug, *PPI* proton pump inhibitor, *PUD* peptic ulcer disease, *TIA* transient ischaemic attack, *VE* vulnerable elder

*McNemar’s test *p* < 0.05

**McNemar’s test *p* < 0.01

***McNemar’s test *p* < 0.001

^a^Prevalence of PPO criteria as a proportion of all participants with the disease or prescribed the drug of interest e.g. prevalence of ACE inhibitor omission as a proportion of all participants who have had an acute MI

^b^Six hundred sixty-one participants (32.2 %) had missing data for measured blood pressure

^c^Hypertension defined using objectively measured blood pressure or self-reported hypertension diagnosis with antihypertensive medication

### Change in prevalence over time

The prevalence of PIMs and PPOs increased significantly (*p* < 0.05) between the baseline and follow-up study periods for each screening tool (Table 1). At follow-up, 64.8 % of participants received a PIM, and 56.6 % received a PPO defined by any of the screening tools, while the proportion of the sample with both a PIM and PPO increased to 41.1 % (843 participants).

The prevalence of PIMs increased between waves and the unadjusted odds ratio (OR) for the presence of any PIM, comparing follow-up to baseline, was 1.08 (95 % CI 1.03, 1.13), using unadjusted GEE analysis. A multivariate GEE model (Table 4) showed that female sex, age and higher number of medicines were significantly associated with change in PIM prevalence and the change in prevalence at follow-up compared to baseline was not significant after adjusting for these covariates. Similarly for PPO prevalence, the association for follow-up compared to baseline in the unadjusted analysis (OR 1.07, 95 % CI 1.02, 1.11) was no longer significant in the multivariable model (Table 4), where age and higher numbers of medicines and chronic conditions were found to be significantly associated with change in PPO prevalence. When included, level of education was not significant in the models and adjusting for it did not alter any of the other odds ratios.

**Table 4:** Population-averaged GEE models for change in sample prevalence of PIMs and PPOs

	Adjusted odds ratio (95 % CI) (*n* = 2046^a^)	
	Any PIM	Any PPO
Follow-up (vs baseline)	1.00 (0.95, 1.06)	0.97(0.92, 1.02)
Age (years)	1.03 (1.02, 1.04)*	1.03 (1.02, 1.04)*
Female (vs male)	1.27 (1.07, 1.5)*	0.86 (0.72, 1.01)
Number of medicines	1.20 (1.17, 1.24)*	1.04 (1.01, 1.07)*
Number of chronic conditions	1.05 (0.99, 1.11)	1.47 (1.39, 1.56)*

*z score *p* < 0.05

^a^Self-reported number of medicines was missing at both time points for five (0.2 %) participants

The most common PIM and PPO criteria (prevalence >2 %) during the follow-up period are presented in Tables 2 and 3, respectively. A number of individual criteria showed highly significant (*p* < 0.0001) increases in prevalence between baseline and follow-up, including prescription of PPIs at full therapeutic dosage for >8 weeks (STOPP, 17.2 to 21.9 %). Only prescription of long-term (>1 month) long-acting benzodiazepines (STOPP/ACOVE, 3.9 to 3.1 %) and omission of antihypertensives in participants with elevated blood pressure (START, 5.5 to 3.5 %) significantly decreased in prevalence.

## Discussion

### Statement of principal findings

This study showed in a cohort of 2051 community-dwelling people aged 65 and over, more than 61 % received a PIM in a 1-year period defined by a subset of STOPP criteria, Beers criteria and ACOVE indicators, while 53 % had a PPO defined by a subset of START criteria and ACOVE indicators. Aspirin for primary prevention, prolonged use of full therapeutic dosage PPIs and strong anticholinergic drugs were the most common PIMs, while common omissions were calcium and vitamin D in osteoporosis, laxatives for patients on opioids and gastro-protection with an NSAID. The increase in PIM and PPO prevalence between baseline and follow-up is associated with patient characteristics (age, female sex, numbers of prescribed medicines and chronic conditions) rather than being a function of time.

### Results in the context of current literature

It is not surprising that the prevalence varies depending on the screening tool used as each includes different types of prescribing in what is classified as potentially inappropriate. Also, the Beers criteria and ACOVE indicators were first developed for use in the USA, whereas STOPP and START are more widely applicable. A number of studies have estimated the prevalence of PIP using multiple screening tools. Prevalence estimates have been highly variable as research has been carried out across settings (hospitals, residential care, community), in a number of countries and using data ranging from full clinical records to administrative data meaning that only a subset of PIP criteria have been applied [17].

A systematic review of studies applying the STOPP and/or START criteria found prevalence ranging from 21 to 79 % for PIMs and 22 to 74 % for PPOs [12]. PIM prevalence according to the Beers criteria varied from 3 to 40 % in studies included in a review of the 1991 Beers criteria, and up to 53.4 % using more recent iterations of Beers criteria [24, 25]. The ACOVE indicators have not been applied extensively, with two studies reporting the prevalence of ACOVE PPOs giving estimates of 58 and 58.5 % [14, 26]. A previous study of TILDA participants using STOPP/START reported lower PIM and PPO prevalence at baseline interview than the current study (14.6 and 30 %) [21]; however, fewer criteria were applied and prevalence was measured at one time point rather than over a period of 12 months.

A number of studies have assessed the prevalence of both PIMs and PPOs; however, the present study appears to be the first to report on the proportion of study participants with concurrent PIMs and PPOs [16–21]. An association has been demonstrated between polypharmacy (using ≥ five medications concomitantly) and underprescribing [27], so the high rate of concurrent PIMs and PPOs is not unexpected.

Few epidemiological studies have reported the longitudinal prevalence of PIP, and findings have shown a trend of PIP decreasing over time [28, 29]. A cohort study which, like the present study, controlled for numbers of prescribed medicines and co-morbidities found that sub-optimal prescribing remained unchanged or decreased over a 4-year follow-up period after adjusting for these factors [30].

### Clinical and policy implications

Potentially inappropriate prescribing in older people is a common issue and warrants attention to improve the quality of care provided to this age group. However, this complex problem may not be accurately captured using administrative data. Participants may not have responded to first-line treatment or have contraindications resulting in a PIM being prescribed or an indicated medicine being omitted. Prescribers may have to weigh up the incremental benefit of one additional indicated medicine against increasing the treatment burden in older patients already taking multiple medications. The structure of the health system in Ireland, in particular the lack of implementation of a co-ordinated chronic disease management policy across primary and secondary care, may also contribute to the rate of PIP, and future implementation of this policy could have a positive impact on prescribing [31].

The strength of evidence of inappropriateness varies across criteria and the risk-benefit ratio may have changed since PIP screening tools were developed. For example, aspirin for primary prevention is included in STOPP, but evidence is conflicting on the net benefit in people with cardiovascular risk factors but without previous cardiovascular events/symptoms [32, 33].

A high proportion of study participants have both prescribing errors of commission (PIMs) and omission (PPOs). This suggests that reviewing both suitability of current medicines and assessing the need for additional indicated therapies is necessary to optimise prescribing for older people.

The long-term prescription of full therapeutic dosage PPIs has been identified previously as a particularly common issue in older people and represents a significant cost burden [34]. Though the cost-effective use of PPIs in Ireland has been promoted through policies such as reference pricing and preferred drug schemes, a focus on prescribing appropriate dosages and durations may provide clinical benefits to patients as well as cost savings [35, 36]. Although long-term benzodiazepine use declined in this study, this may be explained by substitution with Z-drug hypnotics which showed a non-significant increase in prevalence. Omission of antihypertensive therapy also declined at follow-up, which may have been due to participants with high blood pressure at baseline interview being advised to discuss this with their general practitioner.

### Strengths and limitations of the study

This study’s participants are community-dwelling older people from a nationally representative study on ageing, which improves the generalisability of these findings. Although only participants with eligibility to the means-tested GMS scheme were included, a high proportion (73.5 %) of TILDA participants aged over 65 reported GMS eligibility (see Fig. 1).

The use of administrative pharmacy claims data in this study may provide more accurate information on medication exposure than self-reported medication use, although good agreement has been found between such sources [22]. It also allows medication exposure to be determined over a 12-month time period to provide a more accurate assessment of PIM and PPO prevalence, as opposed to using medication data from one point in time which could underestimate PIMs and overestimate PPOs.

A limitation of pharmacy claims data is that patients may not have actually consumed medications dispensed (i.e. if patients are non-adherent), and a lack of information on medicines purchased over-the-counter may lead to an overestimation of some prescribing omissions (e.g. calcium and vitamin D, laxatives). Additionally, there may have been a clinical reason why some participants had a PIM/PPO; however, as no clinical notes were available, it is not possible to determine clinicians’ rationale for such prescribing decisions.

A number of criteria from each of the PIM and PPO screening tools could not be included in this analysis due to the required information not being available in the administrative and survey data used. However, the combination of these data sources allows for a greater number of criteria to be applied than with either source alone [21]. Some information on diagnoses was based on participants self-report and so may not accurately reflect the presence/absence of the conditions of interest.

## Conclusions

Although prevalence of PIMs and PPOs can vary depending on the screening tool used, such prescribing issues are common and may become more prevalent in patients with more medicines or chronic illnesses. This underlines the importance of ongoing prescribing review for older patients, both to assess the appropriateness of current drug therapy as well as to evaluate the need for additional clinically indicated treatments. Further research is planned to examine the association between potentially inappropriate prescribing over time and adverse health outcomes.

## Electronic supplementary material

Below is the link to the electronic supplementary material.

ESM 1 (DOCX 15 kb)

ESM 2 (DOCX 58 kb)
