# Supplementary material for: Longitudinal prevalence of potentially inappropriate medicines and potential prescribing omissions in a cohort of community-dwelling older people
Source: Eur J Clin Pharmacol. 2015 Feb 11;71(4):473–82. doi: 10.1007/s00228-015-1815-1 (PMC4356885; doi:10.1007/s00228-015-1815-1)
Supplement: Supplementary file 1 — (DOCX 15 kb) [file 228_2015_1815_MOESM1_ESM.docx]

**Supplementary file 1** Data collected in TILDA study

**Computer-aided personal interview (CAPI)**

Demographics

Social circumstances

- Transfer to (and from) children
- (Instrumental) activities of daily living and helpers
- Transfers to (and from) parents
- Social connectedness

Health and healthcare

- Physical health
- Cognitive health
- Mental Health
- Behavioural health
- Medications
- Healthcare utilisation

Employment and livelong learning

- Employment situation
  - Current activity status
  - Main job
  - Subsidiary jobs
  - Self-employment
  - Unemployment and permanent sickness/disability
- Job history
- Livelong learning

Planning for retirement and expectations

- Planning for retirement
  - Occupational pensions
  - Public sector pensions
  - Personal retirement savings account
  - Personal pension plans
- Expectations

Sources of income, assets and transport

- Sources of income
- Assets
  - Home ownership
  - Other assets
- Transport

**Self-completion questionnaire**

Social connectedness

Loneliness

Perceived Stress

Stressful life events

Anxiety

Worry

Quality of life

Ageing perceptions

Alcohol

**Health assessment**

Neuropsychological

- Mini mental state examination (MMSE)
- Montreal cognitive assessment (MOCA)
- Sustained attention
- Choice reaction time
- Visual memory
- Executive function

Cardiovascular

- Waist-hip ratio
- Heart rate variability
- Pulse wave velocity
- Phasic blood pressure

Gait, balance and sensory

- Timed up & go
- Gait assessment
- Balance
- Visual acuity & contrast sensitivity

Strength and bone density

- Grip strength
- Hell ultrasound

Macular degeneration

- Macular pigment optical density
- Retinal photograph
