# Supplementary material for: Longitudinal prevalence of potentially inappropriate medicines and potential prescribing omissions in a cohort of community-dwelling older people
Source: Eur J Clin Pharmacol. 2015 Feb 11;71(4):473–82. doi: 10.1007/s00228-015-1815-1 (PMC4356885; doi:10.1007/s00228-015-1815-1)
Supplement: Supplementary file 2 — (DOCX 58 kb) [file 228_2015_1815_MOESM2_ESM.docx]

**Supplementary file 2** Prevalence of individual PIM criteria and PPO criteria at baseline and 2 year follow-up

|  | Baseline | | | Follow-up | | |  |
| --- | --- | --- | --- | --- | --- | --- | --- |
| Criteria description | n | % of sample | % of indication^a^ | n | % of sample | % of indication^a^ | Change in prevalence (95% CI) |
| **PIM criteria** |  |  |  |  |  |  |  |
| **STOPP** |  |  |  |  |  |  |  |
| **Cardiovascular system** |  |  |  |  |  |  |  |
| Digoxin at dose >125 μg/day | 22 | 1.1 | 30.6 | 21 | 1.0 | 23.9 | 0.0 (-0.7, 0.6) |
| Loop diuretic for dependent ankle oedema only | 97 | 4.7 | - | 114 | 5.6 | - | 0.8 (-0.6, 2.2) |
| Loop diuretic as first-line monotherapy for hypertension | 20 | 1.0 | 2.0 | 29 | 1.4 | 2.6 | 0.4 (-0.2, 1.1) |
| Thiazide diuretic with a history of gout | 4 | 0.2 | 5.3 | 3 | 0.1 | 3.7 | 0.0 (-0.3, 0.2) |
| Non-cardioselective β blocker with COPD | 4 | 0.2 | 1.5 | 7 | 0.3 | 2.2 | 0.1 (-0.2, 0.5) |
| β blocker in combination with verapamil | 3 | 0.1 | - | 6 | 0.3 | - | 0.1 (-0.1, 0.4) |
| Aspirin and warfarin in combination without histamine H_2_ receptor antagonist (except Cimetidine) or PPI | 8 | 0.4 | 33.3 | 15 | 0.7 | 30.0 | 0.3 (-0.1, 0.8) |
| Dipyridamole as monotherapy for cardiovascular secondary prevention | 0 | 0.0 | - | 1 | 0.0 | - | 0.0 (0.0, 0.1) |
| Aspirin with history of PUD without H_2_ receptor antagonist or PPI | 40 | 2.0 | 71.4 | 38 | 1.9 | 59.4 | -0.1 (-0.9, 0.7) |
| Aspirin at dose >150 mg/day | 19 | 0.9 | 2.1 | 20 | 1.0 | 2.2 | 0.0 (-0.5, 0.6) |
| Aspirin with no history of coronary, cerebral or peripheral arterial symptoms or occlusive arterial event | 406 | 19.6 | - | 381 | 18.4 | - | -1.2 (-3.9, 1.4) |
| **Central nervous system** |  |  |  |  |  |  |  |
| TCAs with dementia | 1 | 0.0 | 4.8 | 3 | 0.1 | 5.6 | 0.1 (-0.1, 0.3) |
| TCAs with glaucoma | 7 | 0.3 | 7.2 | 6 | 0.3 | 4.7 | 0.0 (-0.4, 0.3) |
| TCAs with cardiac conductive abnormalities | 12 | 0.6 | 5.3 | 20 | 1.0 | 6.6 | 0.4 (-0.2, 0.9) |
| TCAs with an opiate or calcium channel blocker | 46 | 2.2 | - | 69 | 3.4 | - | 1.1 (0.1, 2.1)** |
| Long-term (>1 month), long-acting benzodiazepines | 80 | 3.9 | 34.8 | 64 | 3.1 | 33.0 | -0.8 (-1.9, 0.4)* |
| Long-term (>1 month) neuroleptics | 8 | 0.4 | 6.4 | 11 | 0.5 | 6.2 | 0.1 (-0.3, 0.6) |
| Long-term (>1 month) neuroleptics in those with Parkinsonism | 3 | 0.1 | 8.6 | 3 | 0.1 | 5.9 | 0.0 (-0.2, 0.2) |
| Phenothiazines in patients with epilepsy | 4 | 0.2 | 10.8 | 13 | 0.6 | 24.1 | 0.4 (0.0, 0.8)* |
| Anticholinergics to treat extrapyramidal side-effects of neuroleptics | 4 | 0.2 | 3.2 | 3 | 0.1 | 1.7 | 0.0 (-0.3, 0.2) |
| Prolonged use (>1 week) of first generation antihistamines | 22 | 1.1 | 95.7 | 33 | 1.6 | 89.2 | 0.5 (-0.2, 1.2) |
| **Gastrointestinal System** |  |  |  |  |  |  |  |
| Prochlorperazine or metoclopramide with Parkinsonism | 1 | 0.0 | 2.9 | 4 | 0.2 | 7.1 | 0.1 (-0.1, 0.4) |
| PPI at full therapeutic dosage for >8 weeks | 353 | 17.2 | 42.6 | 450 | 21.9 | 47.4 | 4.7 (2.0, 7.4)*** |
| **Respiratory system** |  |  |  |  |  |  |  |
| Theophylline as monotherapy for COPD | 14 | 0.7 | 5.2 | 11 | 0.5 | 3.5 | -0.1 (-0.6, 0.3) |
| Systemic corticosteroids instead of inhaled corticosteroids in COPD | 5 | 0.2 | 2.1 | 9 | 0.4 | 3.2 | 0.2 (-0.2, 0.6) |
| Nebulised ipratropium with glaucoma | 0 | 0.0 | - | 1 | 0.0 | 0.8 | 0.0 (0.0, 0.1) |
| **Musculoskeletal system** |  |  |  |  |  |  |  |
| NSAID with history of PUD, unless with concurrent H_2_ receptor antagonist, PPI or misoprostol | 37 | 1.8 | 77.1 | 41 | 2.0 | 83.7 | 0.2 (-0.6, 1.0) |
| NSAID with moderate-severe hypertension >160/100 mmHg^b^ | 187 | 9.1 | 33.0 | 207 | 10.1 | 34.0 | 1.0 (-0.9, 2.9) |
| NSAID with heart failure | 14 | 0.7 | 41.2 | 16 | 0.8 | 34.0 | 0.1 (-0.4, 0.6) |
| Long-term use of NSAID (>3 months) | 99 | 4.8 | 14.2 | 107 | 5.2 | 16.2 | 0.4 (-1.0, 1.8) |
| Warfarin and NSAID together | 10 | 0.5 | - | 9 | 0.4 | - | 0.0 (-0.5, 0.4) |
| NSAID with chronic renal failure | 4 | 0.2 | 25.0 | 5 | 0.2 | 26.3 | 0.0 (-0.2, 0.3) |
| Long-term corticosteroids (>3 months) as monotherapy for rheumatoid arthrtitis/osteorarthritis | 37 | 1.8 | 20.6 | 47 | 2.3 | 20.8 | 0.5 (-0.4, 1.4) |
| **Urogenital system** |  |  |  |  |  |  |  |
| Bladder antimuscarinic drugs with dementia | 1 | 0.0 | 4.8 | 5 | 0.2 | 9.3 | 0.2 (0.0, 0.4) |
| Bladder antimuscarinic drugs with chronic glaucoma | 7 | 0.3 | 7.4 | 12 | 0.6 | 9.8 | 0.2 (-0.2, 0.7) |
| α blockers in males with frequent incontinence | 6 | 0.3 | 27.3 | 13 | 0.6 | 39.4 | 0.3 (-0.1, 0.8) |
| **Endocrine system** |  |  |  |  |  |  |  |
| Glibenclamide or chlorpropamide with type 2 diabetes mellitus | 5 | 0.2 | 2.1 | 4 | 0.2 | 1.6 | 0.0 (-0.3, 0.2) |
| Oestrogens with a history of breast cancer | 0 | 0.0 | - | 1 | 0.0 | 1.7 | 0.0 (0.0, 0.1) |
| **Drugs that adversely affect fallers** |  |  |  |  |  |  |  |
| Benzodiazepines in those prone to falls | 63 | 3.1 | 14.4 | 55 | 2.7 | 11.4 | -0.4 (-1.4, 0.6) |
| Neuroleptic drugs in those prone to falls | 34 | 1.7 | 7.8 | 49 | 2.4 | 10.2 | 0.7 (-0.1, 1.6) |
| First generation antihistamines in those prone to falls | 5 | 0.2 | 1.1 | 10 | 0.5 | 2.1 | 0.2 (-0.1, 0.6) |
| Long-term opiates (>1 month) in those with recurrent falls | 8 | 0.4 | 5.1 | 18 | 0.9 | 8.9 | 0.5 (0.0, 1.0) |
| **Analgesic drugs** |  |  |  |  |  |  |  |
| Use of long-term strong opiates as first line therapy for mild-moderate pain | 20 | 1.0 | 29.4 | 18 | 0.9 | 34.0 | -0.1 (-0.7, 0.5) |
| Regular opiates for >2 weeks without concurrent use of laxatives | 109 | 5.3 | 90.8 | 103 | 5.0 | 84.4 | -0.3 (-1.7, 1.1) |
| **Duplicate drug classes** |  |  |  |  |  |  |  |
| Any regular duplicate drug class prescription | 51 | 2.5 | - | 66 | 3.2 | - | 0.7 (-0.3, 1.8) |
| Duplicate antidepressant | 4 | 0.2 | 4.3 | 8 | 0.4 | 6.6 | 0.2 (-0.1, 0.5) |
| Duplicate SSRI | 0 | 0.0 | - | 1 | 0.0 | 0.5 | 0.0 (0.0, 0.1) |
| Duplicate opiate | 22 | 1.1 | 5.3 | 33 | 1.6 | 5.9 | 0.5 (-0.2, 1.2) |
| Duplicate NSAID | 7 | 0.3 | 1.0 | 4 | 0.2 | 0.6 | -0.1 (-0.5, 0.2) |
| Duplicate loop diuretic | 1 | 0.0 | 0.4 | 0 | 0.0 | - | 0.0 (-0.1, 0.0) |
| Duplicate ACE inhibitor | 17 | 0.8 | 2.4 | 20 | 1.0 | 2.7 | 0.1 (-0.4, 0.7) |
|  |  |  |  |  |  |  |  |
| **Beers criteria (2012)** |  |  |  |  |  |  |  |
| **Anticholinergics** |  |  |  |  |  |  |  |
| First-generation antihistamines | 5 | 0.2 | - | 14 | 0.7 | - | 0.4 (0.0, 0.9) |
| Benztropine or trihexyphenidyl | 0 | 0.0 | - | 0 | 0.0 | - | 0.0 (0.0, 0.0) |
| Antispasmodics | 35 | 1.7 | - | 46 | 2.2 | - | 0.5 (-0.3, 1.4) |
| **Antithrombotics** |  |  |  |  |  |  |  |
| Dipyridamole, oral short acting | 7 | 0.3 | - | 10 | 0.5 | - | 0.1 (-0.2, 0.5) |
| Ticlopidine | 0 | 0.0 | - | 0 | 0.0 | - | 0.0 (0.0, 0.0) |
| **Anti-infective** |  |  |  |  |  |  |  |
| Nitrofurantoin (avoid for long-term suppression) | 16 | 0.8 | 27.6 | 35 | 1.7 | 36.5 | 0.9 (0.2, 1.6)** |
| **Cardiovascular** |  |  |  |  |  |  |  |
| α_1_ blockers (doxazosin, prazosin, terazosin), avoid as anti-hypertensives | 14 | 0.7 | 1.4 | 10 | 0.5 | 0.9 | -0.2 (-0.7, 0.3) |
| Central α agonists | 7 | 0.3 | - | 3 | 0.1 | - | -0.2 (-0.5, 0.1) |
| Disopyramide | 2 | 0.1 | - | 0 | 0.0 | - | -0.1 (-0.2, 0.0) |
| Digoxin > 0.125 mg/day | 22 | 1.1 | 30.6 | 21 | 1.0 | 23.9 | 0.0 (-0.7, 0.6) |
| Nifedipine, immediate release | 25 | 1.2 | 100.0 | 24 | 1.2 | 100.0 | 0.0 (-0.7, 0.6) |
| Spironolactone > 25 mg/day | 8 | 0.4 | 25.8 | 8 | 0.4 | 25.0 | 0.0 (-0.4, 0.4) |
| **Central nervous system** |  |  |  |  |  |  |  |
| Tertiary TCAs | 97 | 4.7 | - | 119 | 5.8 | - | 1.1 (-0.3, 2.5)* |
| Thioridazine and mesoridazine | 0 | 0.0 | - | 0 | 0.0 | - | 0.0 (0.0, 0.0) |
| Barbiturates | 3 | 0.1 | - | 3 | 0.1 | - | 0.0 (-0.2, 0.2) |
| Benzodiazepines, short, intermediate and long acting | 201 | 9.8 | - | 180 | 8.8 | - | -1.0 (-2.9, 0.8) |
| Chloral hydrate | 0 | 0.0 | - | 0 | 0.0 | - | 0.0 (0.0, 0.0) |
| Meprobamate | 0 | 0.0 | - | 0 | 0.0 | - | 0.0 (0.0, 0.0) |
| Non-benzodiazepine (Z drug) hypnotics, avoid chronic use >90 days | 48 | 2.3 | 17.3 | 56 | 2.7 | 17.4 | 0.4 (-0.6, 1.4) |
| Ergot mesylates and isoxsuprine | 0 | 0.0 | - | 0 | 0.0 | - | 0.0 (0.0, 0.0) |
| **Endocrine** |  |  |  |  |  |  |  |
| Androgens (methyltestosterone, testosterone) | 3 | 0.1 | - | 1 | 0.0 | - | -0.1 (-0.3, 0.1) |
| Dessicated thyroid | 0 | 0.0 | - | 0 | 0.0 | - | 0.0 (0.0, 0.0) |
| Estrogens with or without progestins | 30 | 1.5 | - | 29 | 1.4 | - | 0.0 (-0.8, 0.7) |
| Growth hormone | 0 | 0.0 | - | 0 | 0.0 | - | 0.0 (0.0, 0.0) |
| Megestrol | 1 | 0.0 | - | 6 | 0.3 | - | 0.2 (0.0, 0.5) |
| Long duration sulfonylureas (chloropropamide, glyburide) | 5 | 0.2 | - | 4 | 0.2 | - | 0.0 (-0.3, 0.2) |
| **Gastrointestinal** |  |  |  |  |  |  |  |
| Metoclopramide | 22 | 1.1 | - | 42 | 2.0 | - | 1.0 (0.2, 1.7)* |
| Mineral oil, oral | 0 | 0.0 | - | 0 | 0.0 | - | 0.0 (0.0, 0.0) |
| **Pain** |  |  |  |  |  |  |  |
| Meperidine | 0 | 0.0 | - | 0 | 0.0 | - | 0.0 (0.0, 0.0) |
| Non-COX-selective NSAIDs, avoid chronic use | 93 | 4.5 | 12.1 | 81 | 3.9 | 10.6 | -0.6 (-1.8, 0.7) |
| Indomethacin and ketorolac | 4 | 0.2 | - | 2 | 0.1 | - | -0.1 (-0.3, 0.1) |
| Pentazocine | 0 | 0.0 | - | 0 | 0.0 | - | 0.0 (0.0, 0.0) |
| Skeletal muscle relaxants | 0 | 0.0 | - | 0 | 0.0 | - | 0.0 (0.0, 0.0) |
| **Drug-Disease interactions** |  |  |  |  |  |  |  |
| Avoid in heart failure (total) | 16 | 0.8 | 47.1 | 18 | 0.9 | 38.3 | 0.1 (-0.5, 0.7) |
| NSAID | 14 | 0.7 | 41.2 | 16 | 0.8 | 34.0 | 0.1 (-0.4, 0.6) |
| Diltiazem | 1 | 0.0 | 2.9 | 1 | 0.0 | 2.1 | 0.0 (-0.1, 0.1) |
| Verapamil | 1 | 0.0 | 2.9 | 1 | 0.0 | 2.1 | 0.0 (-0.1, 0.1) |
| Avoid in epilepsy (total) | 9 | 0.4 | 24.3 | 16 | 0.8 | 29.6 | 0.3 (-0.1, 0.8) |
| Tramadol | 5 | 0.2 | 13.5 | 11 | 0.5 | 20.4 | 0.3 (-0.1, 0.7) |
| Olanzapine | 5 | 0.2 | 13.5 | 5 | 0.2 | 9.3 | 0.0 (-0.3, 0.3) |
| Avoid in dementia (total) | 7 | 0.3 | 33.3 | 27 | 1.3 | 50.0 | 1.0 (0.4, 1.5)*** |
| Anticholinergics | 3 | 0.1 | 14.3 | 10 | 0.5 | 18.5 | 0.3 (0.0, 0.7)* |
| Antipsychotics | 2 | 0.1 | 9.5 | 9 | 0.4 | 16.7 | 0.3 (0.0, 0.7)* |
| Benzodiazepines | 4 | 0.2 | 19.0 | 15 | 0.7 | 27.8 | 0.5 (0.1, 1.0)** |
| H_2_ receptor antagonist | 0 | 0.0 | - | 4 | 0.2 | 7.4 | 0.2 (0.0, 0.4) |
| Avoid with history of falls/fractures (fracture and fall or >1 fall or >1 fracture) (total) | 124 | 6.0 | 54.4 | 168 | 8.2 | 59.2 | 2.1 (0.5, 3.8)** |
| Anticonvulsants | 20 | 1.0 | 8.8 | 46 | 2.2 | 16.2 | 1.3 (0.5, 2.0)*** |
| Antipsychotics | 19 | 0.9 | 8.3 | 31 | 1.5 | 10.9 | 0.6 (-0.1, 1.3) |
| Benzodiazepines | 64 | 3.1 | 28.1 | 76 | 3.7 | 26.8 | 0.6 (-0.5, 1.7) |
| Z drugs | 42 | 2.0 | 18.4 | 60 | 2.9 | 21.1 | 0.9 (-0.1, 1.8)* |
| TCAs | 12 | 0.6 | 5.3 | 27 | 1.3 | 9.5 | 0.7 (0.1, 1.3)* |
| SSRIs | 40 | 2.0 | 17.5 | 45 | 2.2 | 15.8 | 0.2 (-0.6, 1.1) |
| Avoid in Parkinson's (total) | 3 | 0.1 | 8.6 | 9 | 0.4 | 16.1 | 0.3 (0.0, 0.6) |
| Antipsychotics | 3 | 0.1 | 8.6 | 7 | 0.3 | 12.5 | 0.2 (-0.1, 0.5) |
| Metoclopramide | 0 | 0.0 | - | 1 | 0.0 | 1.8 | 0.0 (0.0, 0.1) |
| Prochlorperazine | 1 | 0.0 | 2.9 | 3 | 0.1 | 5.4 | 0.1 (-0.1, 0.3) |
| Promethazine | 0 | 0.0 | - | 2 | 0.1 | 3.6 | 0.1 (0.0, 0.2) |
| Avoid with history of GI ulcers: aspirin >325 mg/d and non COX-2 selective NSAIDs | 28 | 1.4 | 20.6 | 30 | 1.5 | 18.6 | 0.1 (-0.6, 0.8) |
|  |  |  |  |  |  |  |  |
| **ACOVE indicators** |  |  |  |  |  |  |  |
| **Depression** |  |  |  |  |  |  |  |
| If a vulnerable elder( VE) is started on antidepressant medication, then TCAs, MAOIs, BDZs, or stimulants should not be used as first line treatment | 1 | 0.0 | - | 1 | 0.0 | - | 0.0 (-0.1, 0.1) |
| If a VE is taking a SSRI, then an MAOI should not be used for at least 2 weeks after termination of the SSRI | 0 | 0.0 | - | 0 | 0.0 | - | 0.0 (0.0, 0.0) |
| If a VE is taking an MAOI, then do not prescribe interacting medications with or for at least 2 weeks after termination of the MAOI. | 0 | 0.0 | - | 0 | 0.0 | - | 0.0 (0.0, 0.0) |
| **Falls and Mobility Problems** |  |  |  |  |  |  |  |
| If ≥2 falls (or 1 fall with injury) in previous year discontinue benzodiazepine | 43 | 2.1 | 14.9 | 44 | 2.1 | 12.0 | 0.0 (-0.8, 0.9) |
| **Hypertension** |  |  |  |  |  |  |  |
| If a VE has HTN discontinue NSAID or COX-2 inhibitor^b^ | 79 | 3.9 | 7.8 | 80 | 3.9 | 7.1 | 0.0 (-1.2, 1.3) |
| **Ischaemic heart disease** |  |  |  |  |  |  |  |
| If a female VE has IHD then discontinue combination estrogen and progesterone therapy. | 1 | 0.0 | 1.0 | 1 | 0.0 | 0.9 | 0.0 (-0.1, 0.1) |
| **Medication Use** |  |  |  |  |  |  |  |
| Avoid propoxyphene | 0 | 0.0 | - | 0 | 0.0 | - | 0.0 (0.0, 0.0) |
| Discontinue benzodiazepines if taking for >1 month | 80 | 3.9 | 34.8 | 64 | 3.1 | 33.0 | -0.8 (-1.9, 0.4)* |
| Avoid medication with strong anticholinergic effects | 245 | 11.9 | - | 288 | 14.0 | - | 2.1 (-0.1, 4.3)** |
| Avoid barbiturates | 0 | 0.0 | - | 0 | 0.0 | - | 0.0 (0.0, 0.0) |
| Avoid meperidine | 0 | 0.0 | - | 0 | 0.0 | - | 0.0 (0.0, 0.0) |
| If a VE receives ketorolac, do not prescribe for >5 days | 0 | 0.0 | - | 0 | 0.0 | - | 0.0 (0.0, 0.0) |
| Avoid ticlopidine | 0 | 0.0 | - | 0 | 0.0 | - | 0.0 (0.0, 0.0) |
| In iron-deficiency anaemia, prescribe no more than one low-dose oral iron tablet daily | 27 | 1.3 | 20.1 | 43 | 2.1 | 30.3 | 0.8 (0.0, 1.6) |
| **Sleep Disorders** |  |  |  |  |  |  |  |
| If a VE has sleep problems, then avoid drowsy antihistamines | 12 | 0.6 | 1.3 | 22 | 1.1 | 2.6 | 0.5 (-0.1, 1.0) |
| If a VE is chronically (>3 months) taking a benzodiazepine, then discontinue the medication. | 17 | 0.8 | 7.4 | 16 | 0.8 | 8.2 | 0.0 (-0.6, 0.5) |
| **Stroke** |  |  |  |  |  |  |  |
| If a female VE has had a TIA or stroke, discontinue any HRT | 2 | 0.1 | 3.0 | 5 | 0.2 | 5.8 | 0.1 (-0.1, 0.4) |
|  |  |  |  |  |  |  |  |
| **PPO criteria** |  |  |  |  |  |  |  |
| **START** |  |  |  |  |  |  |  |
| **Cardiovascular System** |  |  |  |  |  |  |  |
| Warfarin (or another oral anticoagulant) in the presence of chronic atrial fibrillation | 154 | 7.5 | 67.5 | 194 | 9.3 | 64.2 | 1.8 (0.1, 3.4)*** |
| Aspirin/clopidogrel with a history of atherosclerotic coronary, cerebral or peripheral vascular disease | 47 | 2.3 | 14.2 | 51 | 2.5 | 14.6 | 0.1 (-0.7, 1.0) |
| Antihypertensive therapy where systolic blood pressure >160 mmHg^c^ | 278 | 5.5 | 25.1 | 289 | 3.5 | 24.2 | -2.0 (-3.4, -0.6)*** |
| Statin therapy with a history of coronary, cerebral or peripheral vascular disease | 67 | 3.3 | 20.7 | 82 | 4.0 | 21.8 | 0.7 (-0.3, 1.8)* |
| ACE inhibitor with chronic heart failure | 6 | 0.3 | 2.0 | 12 | 0.6 | 3.2 | 0.3 (0.1, 0.5)* |
| ACE inhibitor following acute myocardial infarction | 61 | 3.0 | 14.6 | 58 | 2.8 | 11.9 | -0.1 (-1.0, 0.8) |
| β blocker with chronic stable angina | 84 | 4.1 | 37.3 | 81 | 3.9 | 33.8 | -0.1 (-1.2, 0.9) |
| **Respiratory System** |  |  |  |  |  |  |  |
| Regular inhaled β_2_ agonist or anticholinergic agent for mild to moderate asthma or COPD | 102 | 5.0 | 37.0 | 116 | 5.7 | 37.5 | 0.7 (-0.6, 2.0) |
| **Central Nervous System** |  |  |  |  |  |  |  |
| L-DOPA in Parkinson’s with definite functional impairment | 0 | 0.0 | 0.0 | 4 | 0.2 | 33.3 | 0.2 (0.2, 0.2) |
| Antidepressant drug in the presence of moderate-severe depressive symptoms | 17 | 0.8 | 42.5 | 11 | 0.5 | 40.7 | -0.3 (-0.6, 0.1) |
| **Musculoskeletal System** |  |  |  |  |  |  |  |
| Bisphosphonates if taking oral corticosteroids for >3 months | 62 | 3.0 | 60.2 | 71 | 3.5 | 64.5 | 0.4 (-0.5, 1.4) |
| Calcium and vitamin D supplement with osteoporosis | 301 | 14.7 | 57.9 | 329 | 16.0 | 51.6 | 1.4 (-0.9, 3.6)* |
| **Endocrine System** |  |  |  |  |  |  |  |
| ACE inhibitor or ARB in diabetes with nephropathy | 5 | 0.2 | 25.0 | 6 | 0.3 | 23.1 | 0.0 (-0.1, 0.2) |
| Antiplatelet therapy in diabetes mellitus if ≥1 major CV risk factor (hypertension, hypercholesterolaemia, smoking history) | 56 | 2.7 | 27.6 | 64 | 3.1 | 28.6 | 0.4 (-0.5, 1.3) |
| Statin therapy in diabetes mellitus if ≥1 major CV risk factor | 50 | 2.4 | 24.6 | 50 | 2.4 | 22.3 | 0.0 (-0.8, 0.8) |
|  |  |  |  |  |  |  |  |
| **ACOVE indicators** |  |  |  |  |  |  |  |
| **COPD** |  |  |  |  |  |  |  |
| If a VE has COPD, prescribe a rapid-acting bronchodilator | 43 | 2.1 | 40.2 | 66 | 3.2 | 46.2 | 1.1 (0.3, 2.0)*** |
| If a VE with COPD has 2+ exacerbations requiring antibiotics/oral corticosteroids in the previous year, then (in addition to a long-acting bronchodilator) prescribe inhaled steroids (if not taking oral steroids) | 20 | 1.0 | 58.8 | 41 | 2.0 | 63.1 | 1.0 (0.4, 1.6)** |
| **Diabetes** |  |  |  |  |  |  |  |
| If a VE with diabetes mellitus has proteinuria, prescribe an ACE inhibitor/ARB | 5 | 0.2 | 25.0 | 6 | 0.3 | 23.1 | 0.0 (-0.1, 0.2) |
| If a VE with diabetes mellitus is not on an anticoagulant or antiplatelet, prescribe daily aspirin | 54 | 2.6 | 26.9 | 58 | 2.8 | 27.2 | 0.2 (-0.7, 1.1) |
| If a VE with diabetes mellitus has LDL-C >130 mg/dL (3.36 mmol/l), prescribe cholesterol-lowering medication^c^ | 3 | 0.2 | 27.3 | 5 | 0.4 | 38.5 | 0.2 (0.0, 0.4) |
| **Hypertension** |  |  |  |  |  |  |  |
| If a VE with HTN has IHD, prescribe a β blocker^b^ | 62 | 3.0 | 37.1 | 63 | 3.1 | 34.6 | 0.0 (-0.9, 1.0) |
| If a VE with HTN has a history of HF, IHD, chronic kidney disease, or CV accident, prescribe an ACE inhibitor/ARB^b^ | 66 | 3.2 | 29.2 | 68 | 3.3 | 26.8 | 0.1 (-0.9, 1.1) |
| **Ischaemic heart disease** |  |  |  |  |  |  |  |
| If a VE with IHD has an LDL-C >100 mg/dL (2.59 mmol/l), prescribe cholesterol-lowering medication^c^ | 15 | 1.1 | 36.6 | 11 | 0.8 | 25.0 | -0.3 (-0.9, 0.3) |
| If a VE with IHD not on warfarin, prescribe aspirin/another antiplatelet | 26 | 1.3 | 12.6 | 25 | 1.2 | 11.8 | 0.0 (-0.6, 0.5) |
| If a VE has had an MI, prescribe a β blocker | 61 | 3.0 | 34.5 | 59 | 2.9 | 30.3 | -0.1 (-1.0, 0.8) |
| If a VE has IHD, prescribe an ACE inhibitor/ARB | 81 | 3.9 | 36.0 | 82 | 4.0 | 34.2 | 0.0 (-1.0, 1.1) |
| **Medication use** |  |  |  |  |  |  |  |
| If a VE with a risk factor for GI bleeding (aged ≥75, PUD, warfarin use, chronic glucocorticoid use) is prescribed a non-selective NSAID, treat concomitantly with misoprostol/a PPI | 207 | 10.1 | 68.5 | 182 | 8.9 | 65.0 | -1.2 (-3.0, 0.5) |
| IF a VE with ≥2 risk factors for GI bleeding is on daily aspirin, treat concomitantly with misoprostol/a PPI | 26 | 1.3 | 41.9 | 32 | 1.6 | 41.6 | 0.3 (-0.3, 0.9) |
| **Osteoporosis** |  |  |  |  |  |  |  |
| If a VE without osteoporosis is taking ≥7.5 mg/d of prednisone (or equivalent) for ≥1 month, prescribe calcium and vitamin D | 53 | 2.6 | 59.6 | 53 | 2.6 | 57.6 | 0.0 (-0.8, 0.8) |
| If a VE without osteoporosis is taking ≥7.5 mg/day prednisone (or equivalent) for ≥3 months, prescribe bisphosphonate therapy | 19 | 0.9 | 48.7 | 23 | 1.1 | 60.5 | 0.2 (-0.3, 0.7) |
| If a VE has osteoporosis, prescribe calcium and vitamin D supplements | 301 | 14.7 | 57.9 | 329 | 16.0 | 51.6 | 1.4 (-0.9, 3.6)* |
| If a female VE has osteoporosis, treat with bisphosphonate, raloxifene, calcitonin, HRT, or teriparatide | 186 | 9.1 | 48.9 | 249 | 12.1 | 53.2 | 3.1 (1.2, 4.9)*** |
| If a male VE has osteoporosis, treat with bisphosphonate, calcitonin, parathyroid hormone, or testosterone | 123 | 6.0 | 87.9 | 144 | 7.0 | 85.2 | 1.0 (-0.4, 2.5)*** |
| **Pain** |  |  |  |  |  |  |  |
| If a VE with persistent pain is treated with opioids, prescribe a stool softener/laxative | 225 | 11.0 | 82.7 | 265 | 12.9 | 82.0 | 2.0 (0.0, 3.9)* |
| **Stroke** |  |  |  |  |  |  |  |
| If a VE has chronic atrial fibrillation and is at medium-high risk for stroke (CHADS2 score ≥2), prescribe anticoagulation | 12 | 0.6 | 9.8 | 19 | 0.9 | 10.8 | 0.3 (0.0, 0.7) |
| If a VE has had a TIA or stroke, prescribe antiplatelet/anticoagulant therapy | 83 | 4.0 | 58.9 | 99 | 4.8 | 54.4 | 0.8 (-0.4, 1.9)** |

Abbreviations: ACE: angiotensin converting enzyme, ARB: angiotensin II receptor blocker, COPD: chronic obstructive pulmonary disease, COX: cyclo-oxygenase, CV: cardiovascular, GI: gastrointestinal, HF: heart failure, HRT: hormone replacement therapy, HTN: hypertension, IHD: ischaemic heart disease, LDL-C: low density lipoprotein cholesterol, MI: myocardial infarction, NSAID: non-steroidal anti-inflammatory drug, PPI: proton pump inhibitor, PUD: peptic ulcer disease, SSRI: selective serotonin reuptake inhibitor, TCA: tricyclic antidepressant, TIA: transient ischaemic attack, VE: vulnerable elder

* McNemar’s test p < 0.05, ** McNemar’s test p < 0.01, *** McNemar’s test p < 0.001

^a^ Prevalence of PIM/PPO criteria as a proportion of all participants with the disease or prescribed the drug of interest e.g. prevalence of benzodiazepines for >4 weeks as a proportion of all participants prescribed a benzodiazepine

^b^ Hypertension defined using objectively measured blood pressure or self-reported hypertension diagnosis with antihypertensive medication

^c^ Missing data on measured blood pressure for 661 (32%) participants and on measured LDL-C for 738 (36%) participants
